# Danhong Injection Price Trend and Its Utilization by Coronary Heart Disease Patients: Evidence From Hospital Records in China

**DOI:** 10.3389/fphar.2022.857167

**Published:** 2022-05-04

**Authors:** Liming Liu, Yue Xu, Zihan Su, Xiaowei Man, Yan Jiang, Liying Zhao, Wei Cheng

**Affiliations:** ^1^ School of Management, Beijing University of Chinese Medicine, Beijing, China; ^2^ Aerospace Center Hospital, Beijing, China; ^3^ National Institute of Chinese Medicine Development and Strategy, BUCM, Beijing, China

**Keywords:** coronary heart disease, Danhong injection, comprehensive reform, association rules, inpatients

## Abstract

**Background:** Danhong injection (DHI) accounts for the highest proportion of drug costs for inpatients with coronary heart disease (CHD). However, if DHI price influences utilization remains unclear.

**Objective:** The objective was to compare changes in the use of DHI for CHD patients during three stages after two comprehensive reforms of public hospitals in Beijing. These findings will provide support for controlling the drug burden of CHD patients and regulating drug use behavior.

**Research Design:** CHD diagnosis and treatment data were extracted from the Hospital Information System (HIS) of 33 public hospitals. Patients were grouped according to different treatment methods and clinical classifications. Changes in the utilization of DHI, including the use rate (the percentage of CHD patients using DHI), number of prescribed units (average number of units of DHI prescribed per hospital stay), and cost of DHI per hospital stay (equal to the unit price multiplied by the number of prescribed units) between the three stages were statistically analyzed. Association rules were applied to identify changes in drug combinations.

**Results:** After the two reforms, the unit price of DHI dropped from $6.46 to $5.61. At the same time, the use rate increased from 20.77 to 24.00%, the number of prescribed units dropped from 29.76 to 29.21, and the cost of DHI per hospital stay dropped from $192.12 to $163.96. The changes in the use rate and number of prescribed units varied among patients with different clinical types and treatment methods, and the cost of CHI per hospital stay was consistent with the overall situation. The variety of drugs used in combination with DHI remained relatively stable.

**Conclusion:** The use rate of DHI for CHD patients increased, indicating increased applications of DHI in clinical practice. Due to the drop in price, the cost of using DHI decreased, and the financial burden of this drug was reduced.

## Introduction

Danhong injection (DHI) is a preparation extracted from the traditional Chinese medicine Danshen and safflower according to a scientific formula that has the function of promoting blood circulation by eliminating blood stasis (Jing et al., 2011). In China, DHI is widely used for the clinical treatment of various cardiovascular and cerebrovascular diseases including stable or unstable angina, acute coronary syndrome, acute myocardial infarction, stroke, transient ischemic attack, and hypertension (Feng et al., 2019; Li et al., 2020). Nevertheless, a previous study found that DHI is the most commonly used traditional Chinese medicine for patients with coronary heart disease (CHD) (Li et al ., 2014).

Before 2009, China’s healthcare system reform focused on setting prices for basic health services with a 15% markup allowed for drug sales. The policy intended to keep basic health services affordable and ensure widespread access, especially for poor farmers. At the time, the government adopted a cost recovery policy in hospital financing, so subsidies to hospitals were greatly reduced. A bonus system was used to reward doctors based on the monetary value of the medicines they prescribed. Thus, financial pressures and the bonus system incentivized physicians to generate revenue for the medical institution and themselves by overprescribing medication (Zhu et al., 2019).

After 2017, China’s medical and health system reform entered a new stage. To reduce the tendency of hospitals to overprescribe drugs, drug markup in public hospitals in China was completely abolished in 2018. Beijing launched two rounds of comprehensive reforms of public hospitals on 8 April 2017 and 15 June 2019. In the first round of reform, 15% of drug markups (except for Chinese medicine decoction pieces) were abolished (Liu et al., 2019). In the second round of reform, the medical consumables markup was eliminated and volume-based procurement (VBP) of drug and medical consumables was implemented (Gao et al., 2021).

Previous studies have demonstrated that drug fees were effectively controlled after these reforms and that the proportion of drug costs of total costs dropped significantly (Liu et al., 2021). While the price of DHI fell after these reforms, few studies have examined the effects of this price change on the use of this drug.

To fill this research gap, this study analyzed changes in the use of DHI by CHD patients before and after the two comprehensive reforms of public hospitals in Beijing. These findings will provide data on controlling the drug burden of CHD patients and regulating drug use behavior.

### Research Hypothesis

We predict that, after the implementation of the reforms leading to the decrease in the price of DHI, the use rate of DHI for CHD patients increased and the cost of DHI per hospital stay decreased.

## Methods

### Data Sources

Data were obtained from the Hospital Information System (HIS) of 33 public hospitals in Beijing, which ensures the authenticity, accuracy, and completeness of the data. Data from the “Medical Record Table,” “Diagnosis Record Table,” and “Charge Details Table” were obtained. The “Medical Record Table” mainly includes basic information such as the patient’s admission date, discharge date, and hospital name. The discharge date was used to determine the stage of the reform when the patient was treated. The “Diagnosis Record Table” includes information related to the patient’s condition and treatment, including main diagnosis, secondary diagnosis, main surgery/operation, and secondary surgery/operation. In this study, the main diagnosis was used to identify CHD patients and the main surgery/operation was used to identify the treatment methods. The “Charge Details Table” includes detailed information about the name of the diagnosis and treatment item, item category, item unit price, and quantity of items used. The name of the diagnosis and treatment item was used to determine whether a patient was treated with DHI.

### Data Processing

First, we extracted the data of patients for who the first three digits of the main diagnosis ICD-10 code ranged from I20 to I25 and whose discharge date was between 1 January 2016 and 31 December 2019. Next, the data were cleaned to delete duplicate records, records with missing key fields, and abnormal data. Lastly, records with a primary diagnosis of CHD were included and any records with a secondary diagnosis were excluded. This resulted in a total of 66,647 medical and diagnosis records and 24,371,139 charge details records being included in the final analysis.

Since there are many clinical types of CHD, only types with a sufficient number of medical records were selected. Unstable angina pectoris (I20.0), acute subendocardial myocardial infarction (I21.4), and arteriosclerotic heart disease (I25.1) had a considerable number of patient records. Thus, these three clinical types were selected for further analysis. Due to large differences in costs, the various treatment types were divided into three categories: medical treatment, percutaneous coronary intervention (PCI), and coronary artery bypass grafting (CABG).

Based on the timing of the hospital stay relative to the implementation of the two hospital reforms, the included patient records were divided into three stages: 1) the pre-reform stage, with discharge dates between 1 January 2016 and 7 April 2016; 2) the first reform stage, with an admission date on or after 8 April 2017 and a discharge date before 14 June 2019; and 3) the second reform stage, with an admission date on or after 15 June 2019 and a discharge date before 31 December 2019.

All cost estimates were converted to United States dollars ($) based on 2019 exchange rates (1 USD = 6.8967 CNY).

### Statistical Analysis

Statistical analysis was performed using Excel software (Microsoft). The use rate, number of prescribed units, and cost of DHI per hospital stay were calculated separately for each of the three stages. The following formulas were used:

Use rate=∑(number of hospitalizations using DHI)/total number of hospitalizations,

Number of prescribed units=∑(number of DHI uses)/total number of hospitalizations,

Cost  of DHI per hospital stay=∑(cost⁡ of DHI use in the hospital)/total number of hospitalizations.

### Association Rules

Association rules are a data mining (DM) method that aims to discover the association relationships between data (Bayardo and Agrawal, 1999). It was first applied to the problem of shopping basket analysis to discover connections between different commodities in consumer transaction data to identify consumer purchase behavior patterns in order to enable better formulation of marketing strategies (Chenguang and Guifa, 2021). In recent years, association rules have also been applied in medical fields (Shi et al., 2019).

Association rules involve the three main indicators of support, confidence, and promotion (Cai, 2020), as detailed in the following.

1) Support represents the probability of item set {X, Y} appearing in the total item set. The formula is

Support(X→Y)=P(X,Y)/P(I)=P(X∪Y)/P(I)=num(X∪Y)/num(I),

where I represents the total transaction set and num () represents the number of occurrences of a specific item set in the transaction set. For example, num (I) represents the number of total transaction sets, while num (X∪Y) represents the number of transaction sets containing {X, Y}.

2) Confidence is the probability that Y is derived from the association rule “X→Y” when prerequisite X occurs. In an item set containing X, the possibility of containing Y is defined by the following formula:

Confidence(X→Y)=P(Y|X)= P(X,Y)/P(X)=P(X∪Y)/P(X).

3) Lift represents the ratio of the probability of including X and the probability of Y occurring at the same time. The formula is as follows:

Lift(X→Y)=P(Y|X)/P(Y).

The effectiveness of an association rule is judged as follows: a rule that satisfies the minimum support and minimum confidence is called a “strong association rule” (the researcher must usually set the parameters in advance). Among strong association rules, there are effective strong association rules and invalid strong association rules. If Lift (X→Y) > 1, the rule “X→Y” is a valid strong association rule; if Lift (X→Y) ≤ 1, then the rule “X→Y” is an invalid strong association rule; and if Lift (X→Y) = 1, this means that X and Y are independent of each other.

In the present study, structured query language (SQL) was used to implement association rules and to identify what medicine(s) co-occurred most frequently with DHI. The conditions of the association rules were as follows: confidence ≥0.8, support ≥0.4, and promotion >1. When more than 10 association rules met these conditions, the first 10 were selected.

## Results

The unit prices of DHI for the three reform stages were $6.46, $5.61, and $5.61, respectively.

**FIGURE 1:**
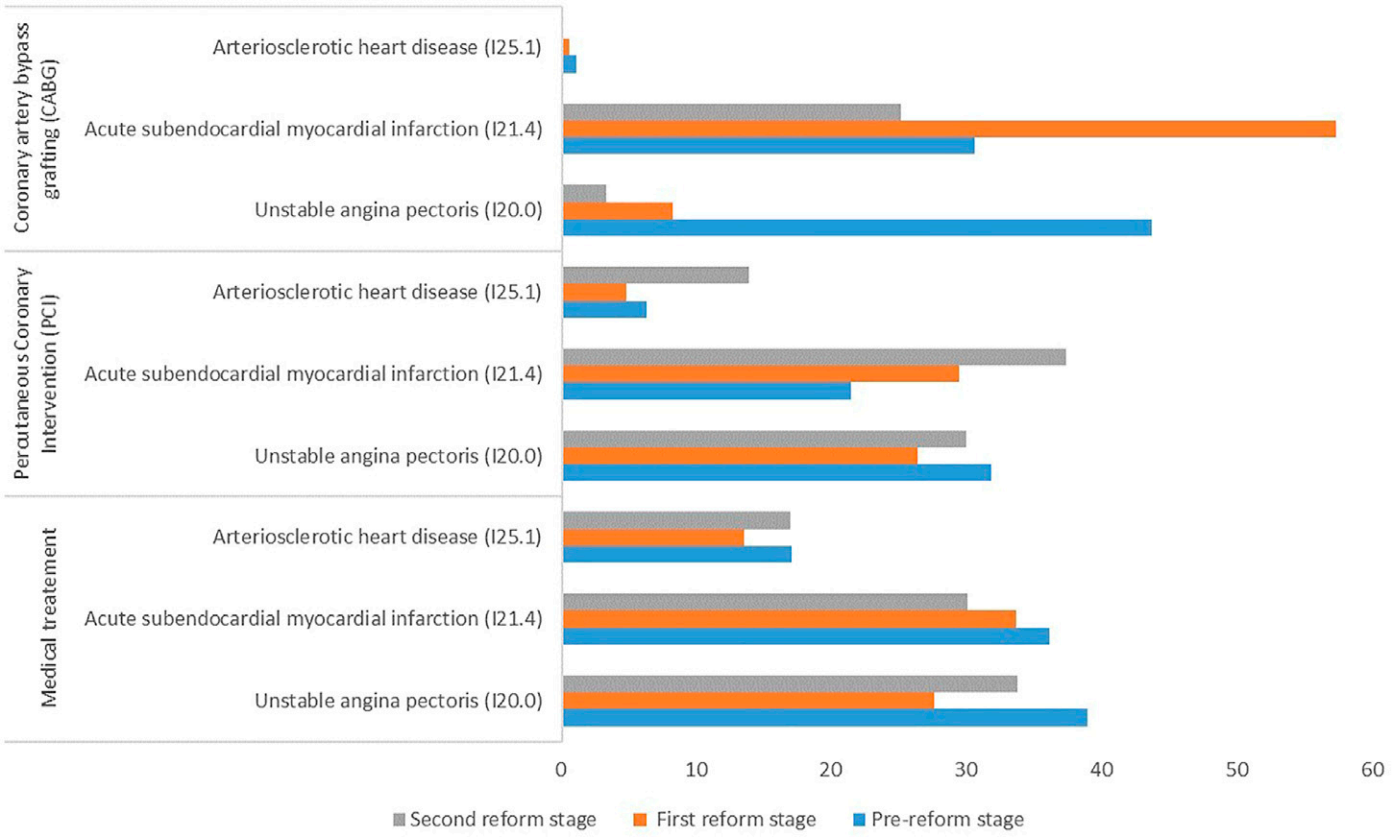
Use rate (%) of DHI among patients with different treatment methods and clinical types of coronary heart disease.

Figure 1 shows the use rate of DHI among inpatients with different clinical types of CHD and treatment methods in the three stages. The use rate of DHI among all included CHD patients increased from 20.77 to 24.00% from the pre-reform stage to the second reform stage. For patients receiving medical treatment, the use rate of DHI among the three CHD types was lower than before the reforms. For patients receiving PCI therapy, the use rate of DHI decreased slightly among I20.0 patients but increased significantly among I21.4 and I25.1 patients. For patients receiving CABG treatment, the use rate of DHI decreased among all three CHD types after the reforms, especially I20.0 patients.

**FIGURE 2:**
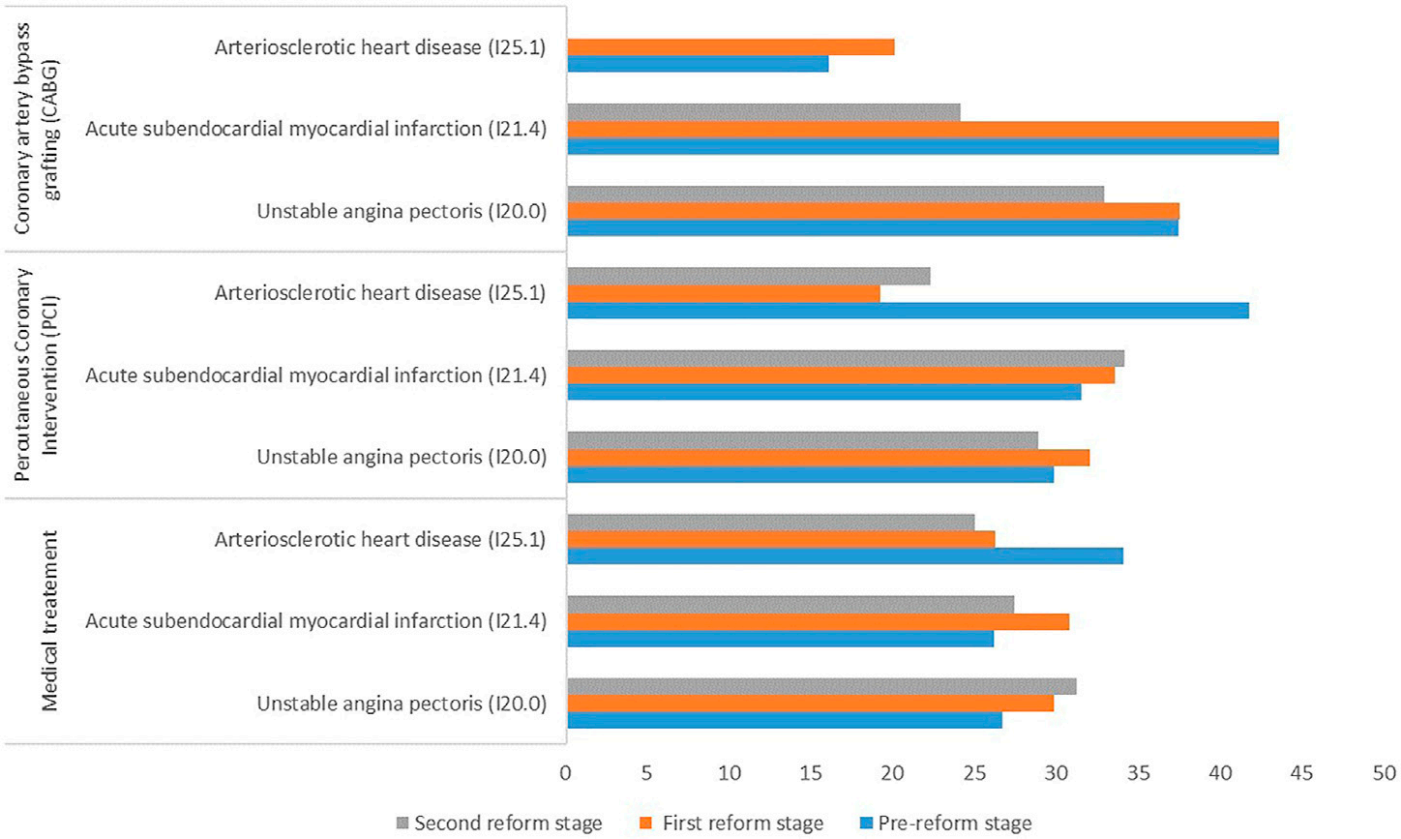
Number of prescribed units of DHI uses among patients with different treatment methods and clinical types of coronary heart disease.

Figure 2 shows the average number of prescribed units of DHI among CHD patients in the three stages. The number of prescribed units of DHI among all CHD patients decreased slightly from 29.76 to 29.21. For patients receiving medical treatment, the number of prescribed units of DHI increased among both I20.0 and I21.4 patients, but decreased among I25.1, compared with before the reforms. For patients receiving PCI treatment, the number of prescribed units of DHI decreased among I20.0 and I25.1 patients but increased among I21.4 patients. For patients receiving CABG treatment, the number of prescribed units of DHI decreased among I20.0 and I21.4 patients.

**FIGURE 3:**
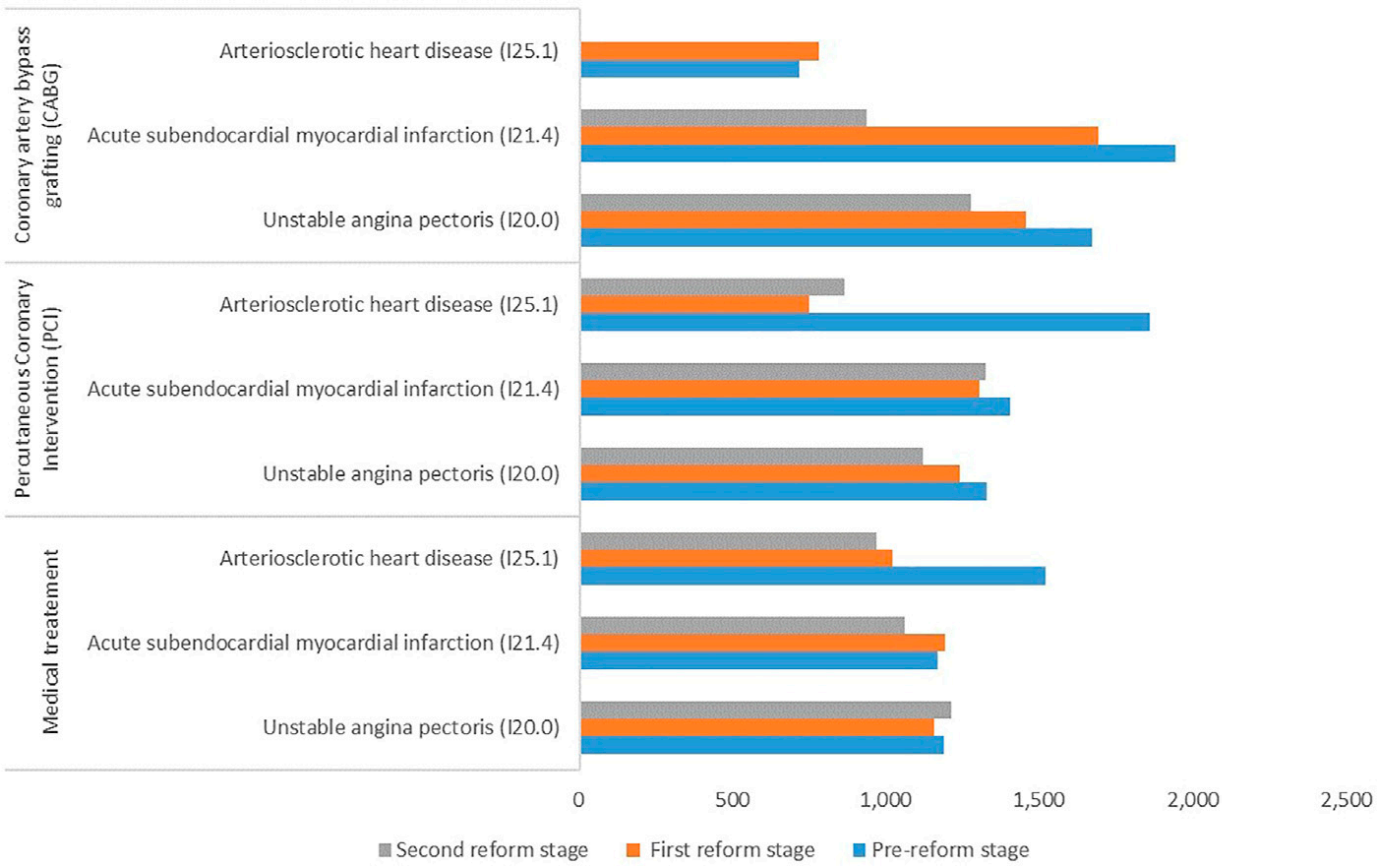
Cost of DHI per hospital stay ($) among patients with different treatment methods and clinical types of coronary heart disease.

Figure 3 shows the average cost of DHI per hospital stay among CHD patients in the three stages. The cost of DHI per hospital stay dropped from $192.12 to $163.96. For patients receiving medical treatment, the cost of DHI per hospital stay increased among I20.0 patients after the reforms but decreased among both I21.4 and I25.1 patients. For patients receiving PCI treatment, the cost of DHI per hospital stay for all three clinical types of CHD patients decreased. For patients receiving CABG treatment, the cost of DHI per hospital stay decreased among both I20.0 and I21.4 patients.

Table 1 shows the association rules and support for the combined use of DHI and another drug in the three stages. After the reforms, the types of drugs used in conjunction with DHI remained relatively stable. They included Western medicines commonly used for the clinical treatment of CHD, such as aspirin, clopidogrel bisulfate, heparin sodium injection, isosorbide mononitrate, nitroglycerin, metoprolol tartrate, and rosuvastatin. There were also dilute solutions commonly used in clinical drugs, such as sodium chloride injection and glucose injection. Among them, sodium chloride injection-DHI consistently showed the highest degree of support, and the probabilities of this drug combination for the three stages were 96.40, 98.58, and 99.04%, respectively. The probability of the combination of trimetazidine hydrochloride and DHI before the reforms was 43.61%, while its probabilities after the two reforms were 40.68 and 33.35%, respectively. Trimetazidine hydrochloride is a cytoprotective drug (Atia et al., 2019) used for the preventative treatment of angina pectoris.

**TABLE 1:** The support of DHI combined with one drug based on association rules.

Pre-reform stage		First reform stage		Second reform stage	
Drug association rules	Support (%)	Drug association rules	Support (%)	Drug association rules	Support (%)
Sodium chloride injection	96.40	Sodium chloride injection	98.58	Sodium chloride injection	99.04
Aspirin	89.60	Aspirin	91.74	Aspirin	92.89
Clopidog hydrogen sulfate	84.03	Clopidogrel bisulfate	85.27	Clopidogrel bisulfate	79.85
Glucose injection	68.73	Nitroglycerin	70.35	Nitroglycerin	69.02
Heparin sodium injection	66.51	Heparin sodium injection	69.18	Heparin sodium injection	62.53
Isosorbide mononitrate	64.41	Isosorbide mononitrate	65.90	Metoprolol tartrate	52.77
Nitroglycerin	63.88	Rosuvastatin	63.32	Isosorbide mononitrate	52.65
Metoprolol tartrate	56.85	Metoprolol tartrate	62.45	Glucose injection	51.47
Rosuvastatin	53.40	Glucose injection	52.68	Rosuvastatin	42.72
Trimetazidine hydrochloride	43.61	Potassium chloride	46.24	Atorvastatin	41.14

Table 2 shows the association rules and support for the combination of two drugs plus DHI in the three stages. The frequency of the “aspirin-sodium chloride injection-DHI” rule was always the highest, and it continued to rise after the reforms with support rising from 86.59 to 92.10%. The “clopidogrel bisulfate-sodium chloride injection-DHI” and “aspirin-clopidogrel bisulfate-DHI” also appeared frequently, but the frequency was lower after the reforms. In general, the two drugs used in combination with DHI have commonly used drugs in clinical practice and the drug association rules were basically unchanged after the reforms, though the support fluctuated slightly.

**TABLE 2:** Supporting degree of DHI combined with two drugs based on association rules.

Pre-reform stage		First reform stage		Second reform stage	
Drug association rules	Support (%)	Drug association rules	Support (%)	Drug association rules	Support (%)
Aspirin–sodium chloride injection	86.59	Aspirin–sodium chloride injection	90.52	Aspirin–sodium chloride injection	92.10
Clopidogrel bisulfate–sodium chloride injection	81.67	Clopidogrel bisulfate–sodium chloride injection	84.43	Clopidogrel bisulfate–sodium chloride injection	79.29
Aspirin–clopidogrel bisulfate	77.39	Aspirin–clopidogrel bisulfate	80.09	Aspirin–clopidogrel bisulfate	76.30
Heparin sodium injection–sodium chloride injection	65.96	Nitroglycerin–sodium chloride injection	70.16	Nitroglycerin–sodium chloride injection	68.85
Glucose injection–sodium chloride injection	65.13	Heparin sodium injection–sodium chloride injection	69.00	Heparin sodium injection–sodium chloride injection	62.47
Nitroglycerin–sodium chloride injection	63.62	Heparin sodium injection–clopidogrel bisulfate	64.66	Heparin sodium injection–nitroglycerin	58.35
Heparin sodium injection–clopidogrel bisulfate	62.79	Rosuvastatin–sodium chloride injection	62.76	Heparin sodium injection–clopidogrel bisulfate	55.25
Glucose injection–clopidogrel bisulfate	59.19	Heparin sodium injection–nitroglycerin	62.26	Metoprolol tartrate–sodium chloride injection	52.26
Heparin sodium injection–nitroglycerin	56.06	Metoprolol tartrate–sodium chloride injection	61.78	Glucose injection–sodium chloride injection	50.51
Metoprolol tartrate–sodium chloride injection	55.21	Metoprolol tartrate–clopidogrel bisulfate	55.21	Metoprolol tartrate–clopidogrel bisulfate	43.74

## Discussion

This study reports the changes in price, use rate, prescribed amount, and cost of DHI per hospital stay among CHD patients and changes in the combined use of drugs after the implementation of the comprehensive drug pricing reforms in China. The results indicate that the policy to abolish drug markup caused the price of DHI to drop significantly after the two rounds of medical reforms. From the perspective of utilization, the utilization rate of DHI increased overall, which is consistent with our hypothesis. This result may be related to the drop in the price of DHI, which increased patients’ demand for higher-priced drugs. Notably, the utilization rates varied for patients with different CHD clinical types and treatment methods. The utilization rate among patients treated with drugs and CABG decreased. For patients receiving PCI treatment, the utilization rate among I20.0 patients decreased while the utilization rate among I21.4 and I25.1 patients increased. This result indicates that the overall increase in DHI utilization rate is mainly due to I21.4 and I25.1 patients also receiving PCI.

The outcome of the number of prescribed units indicated that the average number of DHI uses remained basically unchanged pre- to post-medical reform. This suggests that there was no overuse of DHI by medical institutions or inpatients after the price drop. This is because medical institutions could no longer profit from drug markup after markup was abolished no matter how many drugs are prescribed. Therefore, the reform policy has had a positive effect on regulating the behavior of medical institutions. However, the number of units prescribed for patients with different clinical types and treatments varied. The number of prescribed units of DHI among I20.0 and I21.4 patients receiving drug therapy increased, while the number of prescribed units of DHI among patients receiving CABG treatment decreased. The number of prescribed units of DHI also decreased among I25.1 patients treated with medication and PCI.

From the perspective of average cost over time, after two rounds of medical reforms, the cost of DHI per hospital stay continued to decline, in line with the hypothesis. As mentioned earlier, the number of prescribed units did not change much. Therefore, we speculate that the decrease in the cost of DHI is mainly related to the decrease in the price of DHI. This finding further illustrates that the elimination of drug markup not only reduces the unit price of DHI but also reduces the overall cost of hospital use of DHI. This proves that the two comprehensive reforms had a positive effect on reducing the cost burden of DHI treatment. In terms of different clinical types and treatment methods, the cost of DHI per hospital stay for most patients decreased compared to before the reforms, which is consistent with the overall situation.

The results of combination therapy showed that when one or two drugs are used in combination with DHI, the types of drugs remained steady after the reforms, indicating that the reforms had little effect on the types of drugs used in combination with DHI. However, the frequency of the combined drugs showed a slight fluctuation, which may be related to changes in the use rate of DHI. Among the drug combinations, the frequency of both “sodium chloride injection-DHI” and “aspirin-sodium chloride injection-DHI” rules increased. This may have occurred because the reforms changed the use rate of DHI, which also affected the frequency of combination therapy.

## Limitation

Due to data limitations, this study was not able to explore economic indicators such as the cost-effectiveness of the drug. Although the cost of DHI has been discussed by some scholars (Yang et al., 2020), there is limited economic evaluation research of traditional Chinese medicine and the quality of such studies is generally low (Yang et al., 2021). Further research on pharmaceutical economics is, thus, warranted.

## Conclusion

This study examined changes in the use of DHI for CHD patients treated in public hospitals in Beijing after comprehensive drug pricing reforms. The results indicated that the use rate of DHI increased overall, indicating increased applications of DHI in clinical practice to a certain extent. In addition, due to the drop in price, the cost of using DHI decreased, and the financial burden of this drug was reduced.

## Data Availability

The original contributions presented in the study are included in the article/Supplementary Material, further inquiries can be directed to the corresponding author.

## References

[B1] Atia N. N. Tawfeek H. M. Rageh A. H. El-Zahry M. R. Abdelfattah A. Younis M. A. (2019). Novel Sublingual Tablets of Atorvastatin calcium/Trimetazidine Hydrochloride Combination; HPTLC Quantification, *In Vitro* Formulation and Characterization. Saudi Pharm. J. 27 (4), 540–549. 10.1016/j.jsps.2019.02.001 31061623 PMC6488851

[B2] Bayardo R. J. Agrawal R. (1999). Mining the most interesting rules. In Proceedings of the fifth ACM SIGKDD international conference on Knowledge discovery and data mining (KDD ’99). New York, NY, USA: Association for Computing Machinery 145–154. 10.1145/312129.312219

[B3] Cai Q. (2020). Cause Analysis of Traffic Accidents on Urban Roads Based on an Improved Association Rule Mining Algorithm. IEEE Access 8, 75607. 10.1109/access.2020.2988288

[B4] Chenguang Z. Guifa T. (2021). Application of an Improved Association Rule Algorithm in Rural Development Assessment in China. IOP Conf. Ser. Earth Environ. Sci. 772 (1). 10.1088/1755-1315/772/1/012089

[B5] Feng X. Li Y. Wang Y. Li L. Little P. J. Xu S. W. (2019). Danhong Injection in Cardiovascular and Cerebrovascular Diseases: Pharmacological Actions, Molecular Mechanisms, and Therapeutic Potential. Pharmacol. Res. 139, 62–75. 10.1016/j.phrs.2018.11.006 30408571

[B6] Gao L. Shi L. Meng Q. Kong X. Guo M. Lu F. (2021). Effect of Healthcare System Reforms on Public Hospitals' Revenue Structures: Evidence from Beijing, China. Soc. Sci. Med. 283, 114210. 10.1016/j.socscimed.2021.114210 34274783

[B7] Jing D. Wei Y. Danhui Y. Xie Y. Yang W. Zhuang Y. (2011). Analysis of Using Danhong Injection to Treatment Coronary Heart Disease Patients Medicines Based on Real World HIS Database. Zhongguo Zhong yao Za Zhi China J. Chin. materia Med. 36 (20), 2821. 10.4268/cjcmm20112017 22292375

[B8] Li G. H. Jiang H. Y. Xie Y. M. Jiang J. J. Yang W. Zhao W. (2014). Analysis of Traditional Chinese Medicine Syndrome, Traditional Chinese Medicine and Western Medicine in 84 697 Patients with Coronary Heart Disease Based on Big Data. Zhongguo Zhong Yao Za Zhi 39 (18), 3462–3468. 10.4268/cjcmm20141809 25532378

[B9] Li S. Duan S. Ning Y. Zhang H. Zheng Q. (2020). Efficacy and Safety of Danhong Injection on Endothelial Function and Inflammatory Factors after the Percutaneous Coronary Intervention for Coronary Heart Disease: A Protocol of Systematic Review and Meta-Analysis of Randomized Controlled Trials. Medicine (Baltimore) 99 (27), e20783. 10.1097/MD.0000000000020783 32629660 PMC7337598

[B10] Liu L. Xu Y. Jiang Y. Zhao L. Yin X. Shen C. (2021). Impact of Beijing Healthcare Reform on the Curative Care Expenditure of Outpatients with Noncommunicable Diseases Based on SHA2011 and Interrupted Time Series Analysis. BMC Health Serv. Res. 21 (1), 1045. 10.1186/s12913-021-07059-y 34600531 PMC8487539

[B11] Liu X. Xu J. Yuan B. Ma X. Fang H. Meng Q. (2019). Containing Medical Expenditure: Lessons from Reform of Beijing Public Hospitals. Bmj 365, l2369. 10.1136/bmj.l2369 31227508 PMC6598718

[B12] Shi H. Zhang L. Zheng L. Liu G. (2019). Research on Data Mining in Medical Data Visualization. Henan, China: Luoyang.

[B13] Yang L. Chen X. Oi Lam Ung C. Zhu H. Hu H. Han S. (2020). Clinical and Economic Evaluation of Salvianolate Injection for Coronary Heart Disease: A Retrospective Study Based on National Health Insurance Data in China. Front. Pharmacol. 11, 887. 10.3389/fphar.2020.00887 32625090 PMC7314915

[B14] Yang N. Zhang H. Deng T. Guo J. J. Hu M. (2021). Systematic Review and Quality Evaluation of Pharmacoeconomic Studies on Traditional Chinese Medicines. Front. Public Health 9, 706366. 10.3389/fpubh.2021.706366 34414159 PMC8368976

[B15] Zhu D. Shi X. Nicholas S. Bai Q. He P. (2019). Impact of China's Healthcare price Reforms on Traditional Chinese Medicine Public Hospitals in Beijing: an Interrupted Time-Series Study. BMJ Open 9 (8), e029646. 10.1136/bmjopen-2019-029646 PMC6701667 31401602

